# Diagnostic value of a nanopore sequencing assay of bronchoalveolar lavage fluid in pulmonary tuberculosis

**DOI:** 10.1186/s12890-023-02337-3

**Published:** 2023-03-08

**Authors:** Zhifeng Liu, Yang Yang, Qingfeng Wang, Lei Wang, Wenjuan Nie, Naihui Chu

**Affiliations:** 1 Beijing Emercency Mecial Center, Beijing, 100031 People’s Republic of China; 2 Tuberculosis Department, Beijing Chest Hospital Affiliated to Capital Medical University, No 9, Beiguan Street, Tongzhou District, Beijing, 101149 People’s Republic of China; 3 Tuberculosis Department, Dezhou Second People’s Hospital, Textile Street, Canal Economic Development Zone, Dezhou, 253007 People’s Republic of China

**Keywords:** Nanopore sequencing assay, Pulmonary tuberculosis, Sensitivity, Specificity

## Abstract

**Background:**

To determine the diagnostic accuracy of a nanopore sequencing assay of PCR products from a M. tuberculosis complex-specific region for testing of bronchoalveolar lavage fluid (BALF) samples or sputum samples from suspected pulmonary tuberculosis (PTB) patients and compare the results to results obtained for MGIT and Xpert assays.

**Methods:**

Cases with suspected PTB (n = 55) were diagnosed from January 2019 to December 2021 based on results of nanopore sequencing, MGIT culture, and Xpert MTB/RIF testing of BALF and sputum samples collected during hospitalization. Diagnostic accuracies of assays were compared.

**Results:**

Ultimately, data from 29 PTB patients and 26 non-PTB cases were analyzed. PTB diagnostic sensitivities of MGIT, Xpert MTB/RIF, and nanopore sequencing assays were 48.28%, 41.38%, and 75.86%, respectively, thus demonstrating that nanopore sequencing provided greater sensitivity than was provided by MGIT culture and Xpert assays (*P* < 0.05). PTB diagnostic specificities of the respective assays were 65.38%, 100%, and 80.77%, which corresponded with kappa coefficient (κ) values of 0.14, 0.40, and 0.56, respectively. These results indicate that nanopore sequencing provided superior overall performance as compared to Xpert and MGIT culture assays and provided significantly greater PTB diagnostic accuracy than Xpert and sensitivity comparable to that of the MGIT culture assay.

**Conclusion:**

Our findings suggest that improved detection of PTB in suspected cases was achieved using nanopore sequencing-based testing of BALF or sputum samples than was achieved using Xpert and MGIT culture-based assays, and nanopore sequencing results alone cannot be used to rule out PTB.

## Background

Tuberculosis (TB) remains a major health problem that afflicts about 10 million people worldwide, as reported by the World Health Organization (WHO) [1]. Although pulmonary tuberculosis (PTB) cases account for only 8.5% of global estimated TB incident cases, PTB is the most common form of TB in China. Importantly, early PTB diagnosis and treatment initiation are necessary to prevent disease progression and reduce high PTB mortality and morbidity rates.

Over the last decade, standard PTB diagnostic methods have mainly included culture-based and Xpert-based assays. Although the sensitivity of mycobacterial growth indicator tube (MGIT) culture testing of bronchoalveolar lavage fluid (BALF) samples or sputum samples is at most 50%, MGIT culture is still the most widely used PTB diagnostic method. However, this method requires a long incubation period and thus cannot provide timely results, prompting researchers to develop molecular tests that can produce results in only a few hours. One such test, the Xpert MTB/RIF (Cepheid) assay or the LAMP method transcription reverse transcription concerted reaction (TRC), are rapid, automated, cartridge-based nucleic acid amplification-driven test that was recommended as an initial microbial diagnostic test for PTB [2]. In 25 previous studies, the pooled sensitivity and specificity of the diagnosis of tuberculosis were 93% and 94% for LAMP, and 89% and 98% for Xpert MTB/RIF [3].

New technologies, such as nanopore sequencing, have significantly lowered cost outlays and reduced run times by an order of magnitude, with nanopore sequencing assays achieving rapid detection of microorganisms through analysis of thousands to billions of independently and simultaneously sequenced DNA fragments. Indeed, as compared with MGIT culture and Xpert assays, nanopore sequencing-based assays are faster, more accurate, and more suitable for high-throughput sample processing [4]. But this previous study only focus on the sputum samples from the paticipants. In this study, we analyzed the diagnostic accuracy of a nanopore sequencing assay of PCR products from a M. tuberculosis complex-specific region to detect mycobacterial pathogenic organisms in BALF and sputum samples obtained from suspected PTB patients then compared the results to MGIT culture and Xpert MTB/RIF assay results.

## Methods

### Patient enrollment

All methods were carried out in accordance with guidelines and regulations of Beijing Chest Hospital. Informed consent was obtained from all subjects or their legal guardian. Patients with suspected PTB (n = 55) seeking treatment at Beijing Chest Hospital from November of 2021 to March 2022 were diagnosed based on testing of BALF and sputum samples collected during hospitalization. All enrolled patients were required to meet at least one of the following criteria: (1) exhibit TB disease symptoms, such as subacute cough, fever, night sweats, and/or weight loss; (2) present with chest X-ray findings including miliary pulmonary nodules or patchy shadows.

Clinicians diagnosed PTB cases according to approved clinical guidelines of the Ministry of Health of the People’s Republic of China. Diagnosed PTB cases complied with one or more of the following criteria: (1) positive microbiological results (including acid-fast smear staining results or culture of *Mycobacteria tuberculosis* from BALF specimens); (2) pathological lung tissue biopsy results consistent with pathological features of TB; (3) reduced lung lesion size or disappearance of lesion(s) after 3 months of anti-TB treatment. Otherwise, cases were classified as non-PTB cases. Non-PTB cases were treated with antibiotics such as penicillin and cephalosporins and were followed up for at least 3 months.

### Clinical specimens

A total of 55 BALF specimens were collected from patients aged 15–85 years who were diagnosed with PTB at Beijing Chest Hospital between November 2021 and March 2022, including 25 and 30 specimens collected from men and women, respectively. All specimens were stored at − 70 °C until needed for DNA extraction.

### DNA extraction

Genomic DNA (gDNA) was extracted from clinical specimens according to an accepted, established method as previously reported [5, 6]. Before initiation of library preparation, gDNA samples were quantified using a Qubit 4.0 fluorometer (Life Technologies, USA) then the purity of each preparation was estimated using a Nanodrop spectrophotometer (Thermo Fisher Scientific, USA). Quality requirements included an OD 260/280 ratio of approximately 1.8 and an OD 260/230 value within the range of 2.0–2.2.

### Polymerase chain reaction (PCR)

We targeted the amplification of four gene sequences for the identification of Mycobacterium tuberculosis, namely *IS6110*, *rpoB*, *hsp65* and *gyrB*. The resistance-determining region of the rpoB gene is primarily responsible for rifampicin resistance and also the species identification of Mycobacterium tuberculosis, so our primers cover this region. These sequencing results that consistent with drug resistance, will guide the patient’s treatment. The regions of these genes were presented in Table 1. For analysis of the 55 study samples, we routinely prepared the PCR mix as directed by the instructions provided with the LongAmp Taq 2 × Master Mix Kit (#M0287, NEB). The 30-μL PCR reaction mix for each sample included 5 μL each of 10 μM forward and reverse primers (see Table 1), 20 ng of gDNA extract, and 15 μL LongAmp Taq 2 × Master mix. The PCR cycling profile consisted of 94 °C for 2 min followed by 30 cycles of 94 °C for 15 s, 60 °C for 15 s, and 68 °C for 40 s and a final 4 °C hold. PCR products for each sample were quantified within the range of approximately 100–200 fmol using gradient dilution. Equimolar amounts of PCR products were mixed then a portion of each mixture was sent to ShengTing Bioinformatics Institute for sequencing.

**Table 1:** Information of primers used for PCR amplification

Gene	Primer ID	Sequence	Size (bp)
IS6110	IS6110-For	CTGAACCGGATCGATGTGTA	982
	IS6110-Re	GGTGGTTCATCGAGGAGGTA	
rpoB	rpoB-For	TGTTGGACATCTACCGCAAG	926
	rpoB-Re	CGAGACGTCCATGTAGTCCA	
hsp65	hsp65-For	TCGAGACCAAGGAGCAGATT	1051
	hsp65-Re	GCGAGCAGATCCTCGTAGAC	
gyrB	gyrB-For	CGAAACCACGGAATACGACT	1157
	hsp65-Re	GTTGTGCCAAAAACACATGC	

### Nanopore library preparation and sequencing

Multiplex PCR amplicons of the 55 study samples were prepared using a Ligation Sequencing Kit (SQK-LSK109; ONT, Oxford, UK) and Native Barcoding Kit (EXPNBD104 and EXP-NBD114; ONT). End-prep and native barcode ligation to amplicons were performed for approximately 3 h using a 100–200-fmol sample diluted in 65 μL of nuclease-free water according to the Native Barcoding Kit protocol. Thereafter, adapter ligation and cleaning steps were performed using a NEB ligation kit and Agencourt AMPure XP beads (Beckman Coulter, USA), respectively, to generate a final adapter-ligated DNA library containing 50–100 fmol of DNA. The library was loaded into an R9.4 flow cell (ONT) containing a sufficient number of effective pores (≥ 800 pores) then DNA sequencing was conducted using a GridION instrument (ONT). After the sequencing run was complete, the flow cell was cleaned using a Flow Cell Wash Kit (EXP-WSH004; ONT) according to the manufacturer’s protocol and stored at 4 °C for later use.

### Nanopore data analysis

Nanopore raw data (fast5) were analyzed using Guppy Version 4.5.2 software (ONT) [4]. Basecalling of data was repeated using the parameter "--config dna_r9.4.1_450 bps_hac.cfg--num_callers 4 --cpu_threads_per_caller 4" then barcode recognition was conducted using the parameter "--barcode_kits EXPNBD104 EXP-NBD114" followed by trimming of sequences using the parameter" “--config configuration.cfg--trim_barcodes”. Thereafter, sequence data were counted using NanoPlot v1.28.1 [7] and variant calls were found using medaka v1.3.2 [8]. Finally, raw reads were mapped to the *Mycobacterium tuberculosis* H37Rv genomic reference sequence then trimmed reads were assembled onto the reference genome using Genomics software.

### Consensus generation for use in gene variation identification

Accuracy of detection of nanopore sequencing variants was determined by aligning assembled nanopore sequences to the Sanger reference sequence using ClustalW. Percent identities were determined for each alignment to ascertain the accuracy of the nanopore sequencing method [9–11].

### Statistical analysis

Continuous variables were expressed as the mean ± SD. 2 × 2 contingency tables were generated to display true positives, true negatives, false negatives, false positives, sensitivity, specificity, positive predictive value (PPV), and negative predictive value (NPV). The paired McNemar Chi-square test was used to compare diagnostic accuracies of the nanopore sequencing assay to corresponding accuracies of MGIT culture and Xpert assays. All statistical analyses were performed using SPSS 24.0, with *P* < 0.05 considered statistically significant.

## Results

### Patients' characteristics

A total of 65 suspected PTB cases who visited the hospital from November 2019 to March 2022 were initially included in our study, of which 10 cases were excluded due to lack of anti-TB treatment outcomes data. Clinical features of the final set of patient enrollees in the study are shown in Table 2. Among the total 55 enrolled cases, 29 cases were diagnosed with PTB and 26 cases were diagnosed with non-PTB diseases, with no differences in average age and gender and sample positivity rate s observed between PTB and non-PTB groups (Table 2). All patients with definite and possible PTB were assigned to the PTB group in order to provide "gold standard" data for use in calculating assay sensitivity and specificity. Among the 26 control patients, 11 were diagnosed with malignant cancers and 15 were diagnosed with other infectious diseases, while all PTB patients and controls were confirmed to be HIV negative. Of the total enrollees, 25 (45.5%) were male. The median age of enrollees was 38.79 ± 15.56 years (range 15–85 years).

**Table 2:** Clinical characteristics of enrolled patients

	Number	PTB	Non-PTB	*P* value
Number of patients	55	29	26	
Age (years) (mean ± SD)		38.79 ± 15.56	45.15 ± 17.78	0.33
Gender				0.32
Male, n (%)	25	15 (51.72)	10 (38.46)	
Female, n (%)	30	14 (48.28)	16 (61.54)	
Sample type				0.46
Bronchoalveolar lavage fluid	31	15	16	
Sputum	24	14	10	
Disease type				
PTB		29		
Malignant cancers			11	
Other infectious diseases			15	

### Nanopore sequencing

All samples were amplified and sequenced successfully using the nanopore sequencing assay workflow, resulting in successful library preparation with an average of 1.6 Mb of total reads per flow cell, 100 kb of sequence mapped to H37Rv, and 85% of reads barcoded. In total, four flow cells were run that all provided similar sequencing results quality using a similar numbers of channel.

To avoid time consuming and tedious sample preparation issues prior to DNA sequencing, we attempted to amplify key genes directly from routine clinical specimens without incorporating an additional DNA purification step. Ultimately, time-to-result analysis of each of the 20 study specimens indicated that the procedure took approximately 15 h (including gDNA extraction, PCR amplification, library preparation, nanopore sequencing, and data analysis). Importantly, the flow cell provided enormous excess capacity for PCR amplicons in our size range. In fact, the 20 sample amplicons were easily sequenced using a flow cell with approximately 100 activated pores to generate complete reads in 3 h with an average total sequence coverage per sample of 20 Mb. Assessments of trimmed barcode sequence data quality from multiplex ONT sequencing experiments using NanoPlot revealed no major differences in sequence output results among the different samples, with sequence quality found to be consistent among the 34 samples, as evidenced by high mean base quality scores within the narrow range of 15.0–15.1.

### PTB diagnostic performance of nanopore sequencing, MGIT culture, and Xpert assays

Comparison of PTB diagnostic performance of the nanopore sequencing assay as compared with performance results for MGIT culture (48.28%) and Xpert assays (41.38%) revealed that the nanopore sequencing assay (75.86) was more sensitive than Xpert (*P* < 0.05) (Table 3) but was not statistically significantly more sensitive than the MGIT assay (75.86% vs. 48.28%, *P* = 0:23). Meanwhile, the specificity of the nanopore sequencing assay was significantly lower than that of the Xpert assay (80.77% vs. 100.00%; *P* < 0.05) (Tables 3 and 4). Furthermore, PPV and NPV obtained using the nanopore sequencing assay were 81.48% and 75.00%, respectively, with respective values of 60.47% and 69.09% obtained via Xpert and values of 60.87% and 53.13%, respectively, obtained via MGIT culture (*P* > 0.05) (Tables 3 and 5). Calculations of kappa coefficients revealed κ values for the nanopore sequencing assay, MGIT culture, and Xpert of 0.56, 0.14, and 0.40, respectively, and respective coincidence rates for the three assays of 78.18%, 56.36%, and 69.09% (Tables 3, 4 and 5).

**Table 3:** Diagnostic performance of nanopore sequencing assay, MGIT culture, and Xpert assay in PTB

	PTB	Non-PTB	Sensitivity	Specificity	PPV	NPV	Coincidence	Kappa
MGIT culture			48.28	65.38	60.87	53.13	56.36	0.14
Positive	14	9						
Negative	15	17						
Xpert			41.38	100	100	60.47	69.09	0.40
Positive	12	0						
Negative	17	26						
Nanopore sequencing assay			75.86	80.77	81.48	75.00	78.18	0.56
Positive	22	5						
Negative	7	21						

*PTB* pulmonary tuberculosis, *Non-PTB* non-pulmonary tuberculosis, *PPV* positive predictive value, *NPV* negative predictive value

**Table 4:** Diagnostic Comparison of nanopore sequencing assay and Xpert assay in PTB

Xpert	Nanopore sequencing assay		Number	*P* value
	Positive	Negative		
Positive	9	3	12	< 0.05
Negative	18	25	43	
Number	27	28	55

**Table 5:** Diagnostic Comparison of nanopore sequencing assay and MGIT culture assay in PTB

MGIT culture	Nanopore sequencing assay		Number	*P* value
	Positive	Negative		
Positive	13	9	22	0.23
Negative	14	19	33	
Number	27	28	55	

**Fig. 1:**
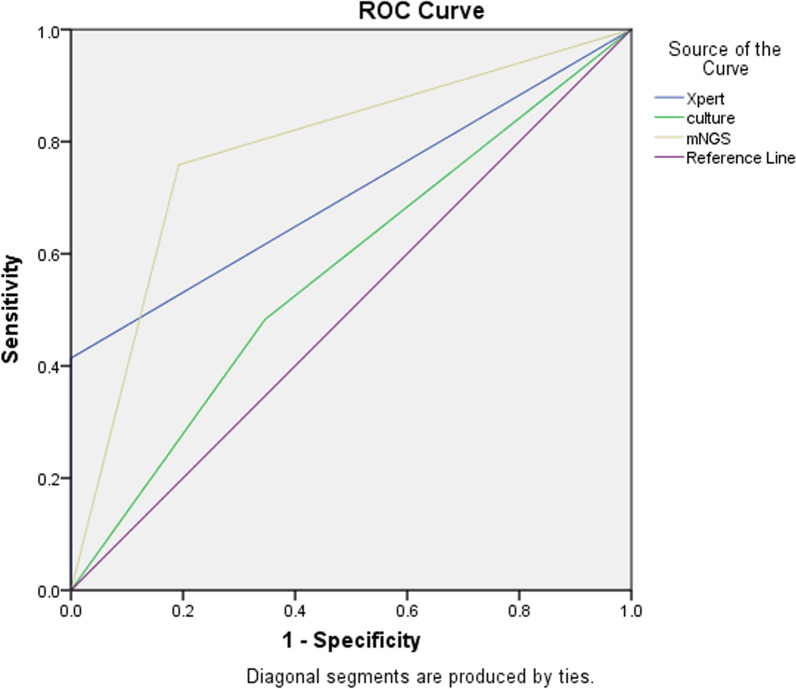
The diagnostic accuracy of different detection techniques

The area under the curve (AUC) value for the nanopore sequencing assay (0.783, 95% CI 0.656, 0.910) was larger than AUC values obtained of 0.568 (95% CI 0.416, 0.721) for the MGIT culture assay and 0.707 (95% CI 0.569, 0.845) for the Xpert assay (Fig. 1). Collectively, these results demonstrate superior diagnostic accuracy of the nanopore sequencing assay as compared to diagnostic accuracies of MGIT culture and Xpert assays when used to diagnose suspected PTB cases.

## Discussion

Subclinical TB infections, known as early-stage active TB disease, are easily ignored, due to a lack of TB symptoms. In order to rule out active TB disease in such cases, radiologic and immunologic assays of sputa or BALF could be used to help detect active disease. However, in cases where patients cannot produce sputa, clinicians are generally reluctant to administer early empirical anti-TB treatments based solely on radiologic findings (e.g., miliary patterns on X-rays) and results of immunologic assays. Therefore, other diagnostic techniques are needed to correctly diagnose TB in sputum-scarce patients with suspected PTB.

Nanopore sequencing assay can accelerate diagnosis of difficult-to-detect pathogens in clinical practice. However, previous studies of nanopore sequencing assay have described excellent nanopore assay-based pathogen detection only in sputum clinical sample. This time we analyze the diagnostic value of a nanopore sequencing assay of bronchoalveolar lavage fluid in pulmonary tuberculosis. When nanopore sequencing assay technologies first became available in the marketplace, they were mainly used for genome sequencing [12]. With the advancement of sequencing chemistries and computational capacity, nanopore sequencing assay technologies have matured into clinical applications in recent years [13]. One such application that is currently used in clinical infectious disease settings, nanopore sequencing, is used most often to diagnose patients experiencing fever without detectable localized infections who lack *M. tuberculosis* culture-positive results [14, 15]. Sequence analysis completion usually required several days, due to sample transport and processing times. In addition, nanopore sequencing assays can support rapid diagnoses of infections caused by slow-growing microorganisms, such as mycobacteria that cause TB and non-tuberculous mycobacterial (NTM) infections [16–21].

Indeed, correct diagnosis of TB versus NTM through rapid identification of mycobacteria at the species level is critically important prior to administration of specific and effective medications in order to maximize treatment outcomes. Moreover, accurate early diagnosis can avoid unnecessary testing and side effects associated with unnecessary medications, thus reducing overall treatment costs. Toward this end, sequence-based assays that only require at most a few days for completion have transformed clinical TB diagnosis, even when samples must be sent to private laboratories for testing. Therefore, use of sequence-based methodologies has undeniably greatly impacted clinical management of mycobacterial infections [4].

The Oxford Nanopore Technologies MinION device is gaining popularity as a platform used by routine clinical microbiology testing laboratories to perform nanopore sequencing assays. When nanopore sequencing assays were first used for laboratory diagnosis of infectious diseases, usually more than 10 samples were processed in one sequencing run in order to reduce the sequencing cost per sample [4]. However, the recent development of the Oxford Nanopore Technologies MinION device has expedited laboratory adoption of diagnostic nanopore sequencing assays, due to its low equipment cost, short turn-around-time, and small physical size for increased portability [7, 8]. When this platform first became commercially available in 2015, its sequencing error rate was still very high [22]. Nevertheless, after several rounds of improvement, the sequencing error rate reached an acceptable range [23, 24] that led to increased demand for the device. In turn, increased demand has fueled further improvements that have led to enhanced scalability, ease of use, and flexibility that have enabled this technology to better serve clinical microbiology laboratories handling diverse specimen volumes. Taken together, these advantages highlight the potential of the Oxford Nanopore Technologies MinION device to serve as an effective platform that will greatly transform clinical microbiological testing [4].

The rapid development of next-generation sequencing has stimulated detection of bacteria, fungi, viruses, and parasitic organisms through nontargeted DNA/RNA sequencing that has facilitated quick identification of pathogens to support early and accurate infectious disease diagnosis. For example, Huang et al. employed a sequence-based assay to successfully detect human pathogens in 94.49% of samples from patients with pulmonary infections who had tested negative for pathogens using traditional pathogen detection methods. Their results thus demonstrated that sequence-based assay accuracy and sensitivity exceeded corresponding standard pathogen detection assay performance indicators [25]. Similarly, Wang et al. found that their sequence-based assay was more sensitive than traditional methods for detection of mixed pulmonary infections [26], whereas Chen et al. obtained sequence-based test results for BALF specimens collected from patients with severe PTB disease that closely aligned with standard culture method-based results [27]. Here we report successful the Nanopore sequence of PCR products from a M. tuberculosis complex-specific region in BALF collected from TB patients and demonstrate its value for use in diagnosing suspected PTB cases. Ultimately, the nanopore sequencing method provided superior sensitivity (75.86%) as compared to culture and Xpert assay sensitivities of 48.28% and 41.38%, respectively. Thus, these results demonstrated greater sensitivity of this method as compared to sensitivities of conventional culture-based and Xpert tests, although its specificity was comparable to that obtained from the conventional tests (e.g., Xpert). Finally, the area under the curve (AUC) value obtained for the nanopore sequencing assay exceeded AUC values obtained for the culture-based assay and Xpert, thus indicating that the nanopore sequencing assay provided superior diagnostic performance when used to test suspected PTB cases. As a final consideration, in cases where one sampling technique is contraindicated, our results revealed that similar results were obtained for sputum and BALF samples, thus demonstrating that clinicians can choose between the two sampling techniques without hesitation. Taken together, these results collectively suggest that nanopore sequencing should be useful as an additional testing method to support improved diagnostic detection of PTB cases.

Although a standard culture-based mycobacterial detection assay was evaluated in this study, this method could not discriminate between TB and NTM organisms as well as Xpert and nanopore sequencing methods, since the culture-based assay produced culture-positive results for both mycobacterial types, as illustrated by the fact that although culture assay specificity was suitable for discriminating between mycobacterium and non-mycobacterium groups, culture assay results obtained for 3 of 29 control samples with NTM disease were-positive for *M. tuberculosis*. Thus, the low specificity of the culture method makes it of limited value when used for TB diagnosis such that the high number of false-positive results obtained using the culture-based method indicate that results obtained using this test should be carefully interpreted when used for TB diagnosis in areas where NTM is epidemic.

Xpert also has limitations in that this assay only targets *rpoB* as a specific *M. tuberculosis* complex-associated sequence, which is present as only a single copy per genome. By contrast, the nanopore sequencing assay targets the IS6110 insertion sequence, which is not only specific for the *M. tuberculosis* complex, but is present at 10–12 copies in genomes of various *M. tuberculosis* strains. Importantly, this feature of nanopore sequencing assays makes them more sensitive than culture-based and Xpert assays for use in detecting diverse clinical *M. tuberculosis* isolates exhibiting variable tissue dissemination patterns and pathogenic properties.

Our study had several limitations. First, the study was based on a relatively small sample size, which might have introduced bias into our results that should be addressed through future studies to evaluate the diagnostic efficacy of the nanopore sequencing assay. Second, since our research focused on BALF specimens, the collection of lavage specimens containing numbers of pathogenic bacteria below the detection level of the test may have resulted in false-negative results as a reminder that the nanopore sequencing assay is still an auxiliary tool that should be used in combination with clinical features, radiological imaging findings, and laboratory test results to diagnose suspected PTB cases. Third, we could not rule out that false-negative and false-positive nanopore sequencing assay results occurred due to (1) a sequence depth that was too low; (2) high host genome background noise and low microbial pathogen biomass; (3) administration of antibiotics to patients prior to testing; and (4) contamination of samples with genomes of environmental microbes or human flora [25]. Fourth, there was an antimicrobial drug resistance gene in our PCR product. But we didn’t have the patients’ treatment result. If these sequencing results consistent with the patient's response to treatment, the correspondence between treatment and sequencing makes our results more beneficial meaningful. Fifth, the absence of certain control diseases, such as lymphomas and rheumatoid arthritis (rare diseases in patients of Beijing Chest Hospital), may have biased our results. However, the limitation may be solved by the combination of Shell-vial culture and nanopore sequencing for biopsy in which the human genome fraction is very important [28].

In summary, our findings demonstrate that nanopore sequencing assay could permit improved detection of *M. tuberculosis* in BALF samples in sputum-scarce cases with suspected PTB. Further investigations are needed to confirm our findings based on larger and more diverse patient populations.

## Conclusions

Our findings suggest that improved detection of PTB in suspected cases was achieved using nanopore sequencing-based testing of BALF or sputum samples than was achieved using Xpert and MGIT culture-based assays, and nanopore sequencing results alone cannot be used to rule out PTB.

## Data Availability

The datasets used or analyzed during the current study are available from the corresponding author on reasonable request. The accession number: PRJNA916099. The link for the database: https://www.ncbi.nlm.nih.gov/bioproject/PRJNA916099/

## Abbreviations

TB: Tuberculosis
BALF: Bronchoalveolar lavage fluid
PTB: Pulmonary tuberculosis
MGIT: Mycobacterial growth indicator tube
gDNA: Genomic DNA
PCR: Polymerase chain reaction
PPV: Positive predictive value
NPV: Negative predictive value
